# Extracellular Vesicles from Mesenchymal Stem Cells as Novel Treatments for Musculoskeletal Diseases

**DOI:** 10.3390/cells9010098

**Published:** 2019-12-31

**Authors:** María José Alcaraz, Alvaro Compañ, María Isabel Guillén

**Affiliations:** 1 Instituto Interuniversitario de Investigación de Reconocimiento Molecular y Desarrollo Tecnológico (IDM), Universitat Politècnica de València, Universitat de València, Av. Vicent A. Estellés s/n, 46100 Burjasot, Valencia, Spain; 2 Department of Pharmacy, Cardenal Herrera-CEU University, Ed. Ciencias de la Salud, 46115 Alfara, Valencia, Spain

**Keywords:** mesenchymal stem cells, extracellular vesicles, immunoregulation, bone diseases, osteoarthritis

## Abstract

Mesenchymal stem/stromal cells (MSCs) represent a promising therapy for musculoskeletal diseases. There is compelling evidence indicating that MSC effects are mainly mediated by paracrine mechanisms and in particular by the secretion of extracellular vesicles (EVs). Many studies have thus suggested that EVs may be an alternative to cell therapy with MSCs in tissue repair. In this review, we summarize the current understanding of MSC EVs actions in preclinical studies of (1) immune regulation and rheumatoid arthritis, (2) bone repair and bone diseases, (3) cartilage repair and osteoarthritis, (4) intervertebral disk degeneration and (5) skeletal muscle and tendon repair. We also discuss the mechanisms underlying these actions and the perspectives of MSC EVs-based strategies for future treatments of musculoskeletal disorders.

## 1. Introduction

Musculoskeletal disorders and injuries are the leading cause of disability worldwide with a high prevalence across the life-course. People with multi-morbidity are particularly vulnerable especially in the context of an aging population. These disorders are painful and lead to mobility limitation and early retirement with a high economic and social impact. They include a wide range of conditions such as osteopenia, osteoporosis, fractures, osteoarthritis (OA), rheumatoid arthritis (RA), sarcopenia and so forth [1].

Transplantation of mesenchymal stem/stromal cells (MSCs) has been recognized in recent years as a promising therapy for musculoskeletal diseases. Preclinical models have provided evidence that MSCs have potential applications in these conditions due to their regenerative and immunomodulatory properties. MSC exhibit a variety of trophic activities relevant to musculoskeletal therapy (reviewed in Reference [2]). The efficacy of MSC treatment has been demonstrated in animal models and clinical studies of RA, OA and cartilage repair [3,4,5,6,7], as well as in bone [8,9], tendon [10] and skeletal muscle [11] regeneration. Although more research is needed including controlled clinical studies with long-term follow-up, these results open the possibility of improving current therapies.

A wide range of evidence has demonstrated that paracrine mechanisms are main components of MSC regenerative effects. MSCs respond to stimuli present in the local microenvironment by secreting a variety of bioactive molecules. Accordingly, it is possible to modulate the composition of the MSC secretome by cellular pre-conditioning during culture, thus maximizing their potential for therapeutic applications (reviewed in Reference [12]). The conditioned medium (CM) from MSCs contains multiple factors that may cooperate to induce a repair response. Besides extracellular vesicles (EVs), MSCs can secrete a wide range of molecules such as purines, bone morphogenetic proteins (BMPs), CD274, C-C motif chemokine ligand (CCL)-2, connexin 43, indoleamine 2,3-dioxygenase, prostaglandin (PG)E_2_, interleukin(IL)-6, IL-10, NO, heme oxygenase-1, tumor necrosis factor-inducible gene-6 (TSG-6), leukemia inhibitory factor (LIF), CD95/CD95 ligand, galectins, human leukocyte antigen-G5 (HLA-G5) and growth factors such as transforming growth factor-β1 (TGF-β1), hepatic growth factor (HGF), vascular endothelial growth factor (VEGF), platelet-derived growth factor, fibroblast growth factor (FGF) and so forth. [2,13,14,15,16,17,18,19]. The use of CM may avoid some problems associated with the therapeutic application of MSCs such as immune rejection of allogeneic cells or undesirable cell differentiation. However, treatment with CM may be an alternative to cellular therapy in regenerative medicine [20].

EVs are main components of MSC secretome which can be incorporated into cells via endocytosis or phagocytosis [21,22,23] leading to the transfer of their content such as proteins, lipids, DNA, RNA, mitochondria and so forth. As a result, EVs regulate gene transcription and the functions of recipient cells [24,25,26,27]. In particular, the transfer of miRNA plays an important role in the biological activity of MSC EVs [28]. Most of the bioactive effects of EVs require the interaction between EV-associated molecules CD9 and CD81 and the binding partners immunoglobulin superfamily, member 8 (IGSF8) and PGF_2_ receptor negative regulator (PTGFRN) on cells [24]. It is widely accepted that EVs represent an important mechanism for cell communication.

EVs are generally classified into exosomes (EXOs, 30–100 nm in diameter, formed by the inward budding of endosomal membrane to produce a multivesicular body that upon fusion with cell membrane releases these microparticles) and microvesicles (MVs, 50 nm to 1000 nm in diameter, generated by the outward budding and fission of the plasma membrane) (reviewed in Reference [29]). Nevertheless, MSCs may secrete other EVs with overlapping size which do not exhibit the same characteristics as EXOs [30]. As currently there are no appropriate methods to isolate pure EV subtypes, better isolation and characterization methods are under study to establish the necessary protocols and classifications needed to know their specific properties allowing the development of EVs for therapeutic applications. To this respect, the International Society for Extracellular Vesicles (ISEV) is committed to improving the standardization guidelines for EV studies [31].

In recent years, there is an increasing interest in the biological activity of EVs which has been demonstrated in many studies highlighting their potential as an alternative to cell therapy with MSCs (for a review, see Reference [32]). MSC EVs can promote angiogenesis and regulate immune responses [33]. In addition, they inhibit apoptosis and increase cell proliferation through extracellular signal-regulated kinase (ERK)1/2 and mitogen-activated protein kinase (MAPK) pathways [34]. EVs contain different adhesion molecules able to interact with cells and extracellular matrix components. EVs also express proteins such as matrix metalloproteinases (MMPs) involved in remodeling processes. The enhancement of cell viability, proliferation, migration and engraftment, vascularization and extracellular matrix formation contribute to the repair activity of MSC EVs [21,35].

A great deal of research has been carried out to find potential applications of MSC EVs. Although there is a broad scope of possible uses (e.g., EV-based drug delivery strategies, as biomarkers of disease, etc.) this review focuses on the current understanding of MSC EVs actions in preclinical studies of musculoskeletal disorders. We also discuss the perspectives to develop future therapeutic treatments based on this approach.

## 2. Immune Regulation and Rheumatoid Arthritis

A wide range of evidence indicates that EVs play an essential role in immunomodulation. EVs may either activate or suppress immune responses depending on the type of parent cell [36,37]. However, many in vitro and in vivo studies have demonstrated the predominant anti-inflammatory and immunosuppressive properties of MSC EVs in both innate and adaptive immune cells [38,39]. These therapeutic effects of EVs may be similar to those of parent cells [40]. EVs contain a number of tolerogenic molecules present in MSCs (programmed death-ligand 1, galectin-1 and TGF-β1) [41]. Nevertheless, some studies have reported a higher immunomodulatory ability of MSCs compared with their EVs which may be mediated by cell contact besides a number of factors present in the secretome [42]. Another study has also shown that B cell modulation by MSCs is partially mediated by soluble factors other than EVs [43]. It has been reported that EVs from human bone marrow MSCs (BM-MSCs) reduce the expression of pro-inflammatory cytokines such as IL-1β, IL-6 and tumor necrosis-α (TNFα) in monocytic cells whereas the expression of anti-inflammatory IL-10 and TGF-β1 is enhanced [44]. The anti-inflammatory properties of EVs can be potentiated by stimulation of MSCs with cytokines such as TNFα+interferon γ [45].

**Figure 1:**
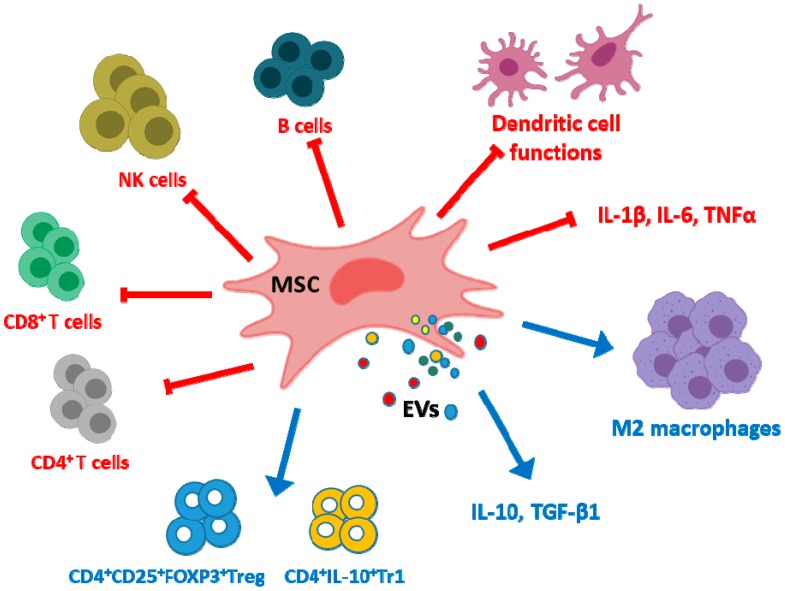
Examples of immunomodulatory effects exerted by mesenchymal stem/stromal cell extracellular vesicles (MSC EVs). EVs inhibit the production of pro-inflammatory cytokines but increase the levels of anti-inflammatory cytokines in monocytic cells. They exert negative effects on dendritic cell maturation and activation with impairment of antigen uptake and also on the proliferation of CD4^+^ T cells, CD8^+^ T cells, NK cells and B cells. These EVs promote the conversion of Th1 into Th2 and reduce Th17 differentiation. In contrast, MSC EVs enhance the differentiation of regulatory cells such as M2 macrophages, CD4^+^CD25^+^FOXP3^+^Treg and CD4^+^IL-10^+^Tr1.

MSC EVs exert immunoregulatory effects by several mechanisms (Figure 1). EVs regulate dendritic cell functions with impairment of antigen uptake by immature cells and reductions in cell maturation and activation [46]. Although there are differences depending on the parent cells, MSC EVs may inhibit the proliferation of CD4^+^ and CD8^+^ T cells and the promotion of apoptosis in CD4^+^ T cells [47,48]. EVs may also promote the conversion of T helper type 1 (Th1) into T helper type 2 (Th2) cells whereas differentiation into Th17 cells was decreased [44]. In addition, EVs polarize activated CD4^+^ T cells to CD4^+^CD25^+^FOXP3^+^ regulatory T cells (Tregs) which requires activation of T cells by antigen presenting cells [47,49,50]. As MSC EVs polarize immune cells toward an immunosuppressive phenotype only in the presence of an activated immune system [50], homeostatic immune activity would not be affected by EVs treatment thus avoiding the increased risk of infection or cancer observed with immunosuppressive drugs [51]. The proliferation and differentiation of B cells and the proliferation of natural killer (NK) cells are reduced by MSC EVs [52,53] while the levels of cytotoxic T lymphocyte-associated protein 4 (CTLA-4), a protein receptor which downregulates immune responses, are enhanced [44]. In particular, EVs have the potential to switch macrophages into an anti-inflammatory phenotype (M2) [54]. Cell pre-conditioning can improve the macrophage differentiation ability of EVs as shown by using hypoxia which increases the content of miRNAs such as miR-223 and miR-146b in EVs [55]. Similarly, treatment of human umbilical cord MSCs (UC-MSCs) with lipopolysaccharide (LPS) has been shown to improve the anti-inflammatory ability of their EXOs through the induction of let-7b which is transferred to recipient cells and targets the toll-like receptor 4 (TLR4)/nuclear factor κ-light-chain-enhancer of activated B cells (NF-κB)/signal transducer and activator of transcription 3 (STAT3)/Akt pathway. As a result, macrophages can be polarized into an anti-inflammatory M2 phenotype [56].

RA is a chronic autoimmune inflammatory disorder characterized by hyperplasia of the synovial membrane and infiltration of immune and inflammatory cells. Synovial cell transformation, excessive production of inflammatory and catabolic mediators and osteoclast generation progressively result in joint destruction and disability [57,58]. Although there is limited information, EVs may represent a novel therapeutic strategy for arthritis. Experimental evidence indicates an immunomodulatory role for MSC EVs which may counteract antigen-driven T cell responses thus suggesting a potential interest of this approach for the treatment of T cell-mediated diseases such as RA. In a model of antigen (bovine serum albumin)-induced synovitis in pigs, intra-articular injection of EVs from pig BM-MSCs into the carpal joint (500 μg protein/injection in 500 μL) significantly reduced lymphocyte counts and gene expression of TNFα in synovial fluid. In addition, gait analysis at day 7 revealed a trend for the improvement of the impulse which may be due to pain reduction linked to the anti-inflammatory effect of EVs [59].

MVs and EXOs from BM-MSCs isolated from C57BL/6 mice did not reduce the proliferation of CD8^+^ or CD4^+^ T lymphocytes but increased CD4^+^CD25^+^FOXP3^+^ Tregs and the CD4^+^IL10^+^ Tr1 regulatory cell population. In the in vivo models of delayed-type hypersensitivity and collagen-induced arthritis (CIA) in mice both EV fractions exerted anti-inflammatory effects and protected joints from degradation, with a higher efficacy of EXOs, which may be related to the inhibition of plasmablast differentiation and the induction of IL-10-expressing regulatory B cells [60].

Annexin A1 is an endogenous inhibitory mediator of arthritis [61] enriched in synovial fluid neutrophil EVs from RA patients [62]. Animal models of RA have shown the role of annexin A1 in the anti-inflammatory and chondroprotective effects of neutrophil-derived EVs which depend on the interaction with its receptor formyl peptide receptor 2 (FPR2), inducing anabolic responses with TGF-β1 production and deposition of extracellular matrix, as well as chondrocyte protection from apoptosis [62]. Interestingly, annexin A1 is a main component of adipose tissue-derived mesenchymal stem cells (AD-MSCs) MVs and may contribute to the anti-inflammatory properties of these EVs [63].

## 3. Bone Repair and Bone Diseases

The secretome of MSCs mediates the mitogenic, pro-migratory and pro-osteogenic effects of these cells promoting bone healing in animal models through the recruitment of MSCs, endothelial cells and progenitor cells [24,64]. It has been reported that EXOs from AD-MSCs enhance the proliferation, migration and differentiation of human osteoblastic cells. These functions are potentiated by TNFα pre-conditioning of AD-MSCs which increases the expression of Wnt-3a in EXOs [65]. BM-MSC EVs promote osteoblast differentiation and expression of osteogenic genes, which is partly mediated by miR-196a as it has been demonstrated in functional experiments [66]. The differentiation stage of progenitor MSCs may influence the miRNA composition of EVs and their ability to induce osteogenic differentiation and mineralization in recipient MSCs [67].

Tissue regeneration requires the recruitment of endogenous stem/progenitor cells to the target site to form tissue specific cells as well as new vessels to provide the necessary oxygen, nutrients and growth factors to repair the affected area [68,69]. The repair effects of EVs on bone have been demonstrated both in vitro and in vivo, in different models of fracture, bone defect or osteoporosis. The relevance of MSC EXOs in bone healing has been shown in a femur fracture model of CD9^-/-^ mice which have impaired EV formation. The retardation of callus formation and fracture healing can be rescued by the injection of BM-MSC EXOs but not by EXOs-free CM [70]. EV administration accelerates bone repair in models of calvarial bone defect in mice and rats. Thus, EVs enhanced osteoblastic proliferation at bone defect edge and stromal progenitor cell migration into the defect mid-substance leading to and increased re-ossification of the defect with increases in bone volume, bone formation area and healing score [24,66]. In addition, EVs from AD-MSCs combined with poly(lactic-co-glycolic acid) (PLGA) scaffolds have demonstrated an enhancing effect on osteogenic, proliferation and migration capabilities of human BM-MSCs in vitro. This approach also enhanced bone regeneration through these osteoinductive effects in critical-sized calvarial defect in mice [71]. Human induced pluripotent stem cells (iPSCs) obtained via genetic reprogramming of adult somatic cells may have some advantages compared with adult MSCs for therapeutic applications. Autologous iPSCs-derived MSCs (iMSCs) have great potential in regenerative medicine and they are superior to MSCs in cell proliferation ability, immunomodulation and secretion of bioactive factors including EVs [72,73]. Therefore, EXOs derived from human iMSCs combined with tricalcium phosphate (β-TCP) improved the osteoinductivity of these scaffolds through activation of phosphatidylinositol-3-kinase (PI3K)/Akt signaling in human BM-MSCs [74].

**Figure 2:**
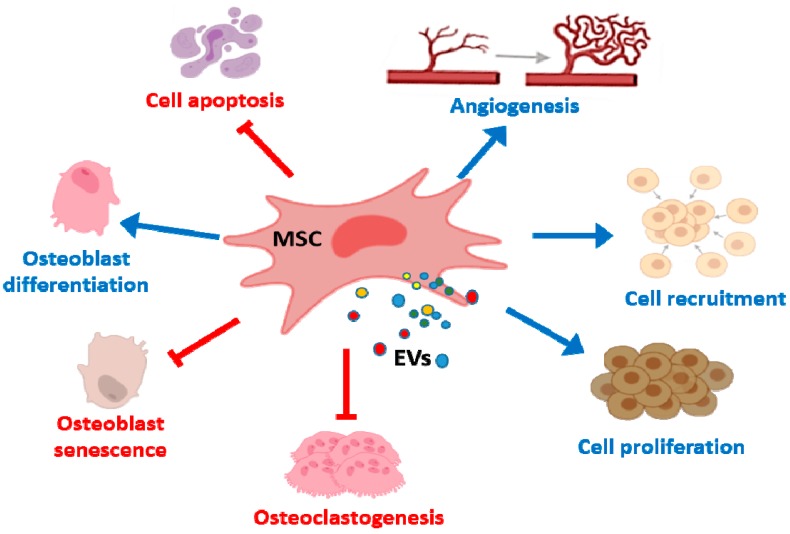
Effects of MSC EVs on bone repair and bone diseases. MSC EVs enhance proliferation, migration and osteogenic differentiation of BM-MSCs whereas apoptosis is inhibited. In addition, proliferation and recruitment of endothelial cells/endothelial progenitor are promoted leading to angiogenesis. MSC EVs also reduce osteoclast formation and osteocyte-like cells apoptosis.

In bone tissue repair, MSC EVs promote osteogenesis and angiogenesis (Figure 2) which may depend on interactions with different cell types such as BM-MSCs and endothelial cells. EVs from MSCs can be useful tools in bone regenerative medicine as they induce lineage specific differentiation of BM-MSCs and can bind to extracellular matrix proteins such as type I collagen and fibronectin [75]. Nevertheless, the source of EVs influences these regenerative properties. Thus, EXOs from diabetic rat BM-MSCs showed a reduced effect on the osteogenic differentiation of BM-MSCs and the angiogenic activity of human umbilical cord endothelial cells, compared with EXOs from healthy rats. In addition, bone regeneration and neovascularization effects in rat calvarial defects were lower for EXOs from diabetic rats [76].

EVs may be provide novel opportunities in endochondral repair of large bone defects which need bone grafts and costly interventions (reviewed in Reference [77]). Thus, EVs may be a better approach compared with cell-based therapies as implanted cells compete with endogenous progenitor cells for oxygen and nutrients in this ischemic microenvironment. In particular, MSCs cannot adapt their glucose consumption and do not possess the necessary glycolytic reserves to maintain their metabolism [78]. In fact, reduction of MSCs metabolic needs by induction of quiescence can enhance their survival under ischemic conditions in the lesion site [79] while hypoxia can reduce their osteogenic potential [80]. In addition, most culture-expanded MSCs cannot adapt to the microenvironment and they die or are phagocytosed by macrophages shortly after implantation in critical-sized bone defects. Distraction osteogenesis induces bone tissue regeneration in large bone defects through the recruitment of endogenous MSCs and endothelial cells/endothelial progenitor cells to the distraction gap. Administration of BM-MSC CM is an effective procedure to accelerate this process as it promotes the migration of Sca-1^+^/PDGFR-α^+^ BM-MSCs which differentiate into osteoblasts and CD31^+^ cells leading to angiogenesis in a mouse model. Soluble factors CCL-2, CCL-5 and CCL-7 can recruit bone marrow mononuclear cells while IL-3 and IL-6 recruit endothelial cells and progenitor cells to enhance osteogenic activity [64]. Administration of EVs from different origins may be another strategy to shorten these lengthy procedures. Recently, it has been demonstrated a role for EXOs from progenitor endothelial cells in the stimulation of angiogenesis thus accelerating bone regeneration during distraction osteogenesis [81].

Besides EVs from BM-MSCs and AD-MSCs, EVs from different MSCs are of interest in bone repair. Therefore, EXOs derived from UC-MSCs enhance fracture healing by promoting angiogenesis in a rat model of stabilized fracture. These EXOs are taken up by endothelial cells leading to hypoxia-inducible factor-1α (HIF-1α) activation and VEGF production with the result of cell proliferation, migration and tube formation [82].

Experimental evidence also indicates the potential application of MSC EVs in osteoporosis. To this respect, implantation of EXOs secreted by iMSCs incorporated in β-TCP scaffolds into critical size calvarial bone defects enhanced osteogenesis and angiogenesis in ovariectomized rats, a model of postmenopausal osteoporosis. In vitro experiments demonstrated the ability of these EVs to stimulate the proliferation and osteogenic differentiation of BM-MSCs from ovariectomized rats [83]. Autologous EVs from human urine-derived stem cells are a potential therapeutic agent for osteoporosis. Systemic injection of these EVs have been shown to promote osteogenesis and inhibit osteoclastogenesis in osteoporotic mice by transferring collagen triple-helix repeat containing 1 (CTHRC1) and osteoprotegerin proteins [84].

Senile osteoporosis may be another potential application of EVs. Therefore, bone loss was attenuated by i.v. administration of EVs from human umbilical cord blood plasma to 16 months old male C57BL/6 mice. This treatment increased trabecular and cortical bone mass and promoted osteoblast differentiation while reducing osteoclast formation. miR-3960 was enriched in these EVs and mediates their osteogenic effects [85]. Increased osteocyte apoptosis and osteoclast activation leads to imbalanced bone remodeling during ageing. AD-MSC EVs reduced hypoxia/serum deprivation-induced apoptosis of osteocyte-like cells through upregulation of Bcl-2/Bax and downregulation of reactive oxygen species (ROS), cytochrome c and activation of caspase-9 and caspase-3. In addition, these EVs reduced osteoclastogenesis suggesting the potential of AD-MSC EVs to improve age-related bone loss [86].

Apoptosis of bone marrow cells plays an important role in the destruction of bone tissue during long-term or high dose glucocorticoid treatment leading to osteonecrosis of the femoral head. Strategies able to prevent apoptosis and promote proliferation of bone marrow cells may be useful to start tissue repair and inhibit disease progression. One of these strategies is the application of MSCs or EVs. Therefore, EXOs derived from human synovial MSCs are internalized by bone marrow cells to promote cell proliferation and inhibition of apoptosis. In a rat model of osteonecrosis of the femoral head induced by methylprednisolone, i.v. administration of 1 × 10^11^ particles of EXOs (in 200 μL) exerted a preventive effect and counteracted the inhibition of the osteogenic response induced by glucocorticoid [87]. It is known that EXOs secreted by human iMSCs promote angiogenesis due to the activation of PI3K/Akt signaling in endothelial cells. As a consequence, i.v. administration in the steroid-induced rat osteonecrosis model prevented bone loss and osteonecrosis of the femoral head [88]. As HIF-1α plays a role in osteogenesis and angiogenesis [89,90], strategies directed at the modulation of this transcription factor may improve the bone repair properties of EVs. Therefore, BM-MSCs transfected with a mutant HIF-1α which is not degraded in normoxic conditions were used to obtain mutant EVs which exhibited enhanced osteogenesis and angiogenesis properties leading to the repair of steroid-induced avascular necrosis of the femoral head in rabbits [91].

Potential therapeutic applications of MSCs from a variety of sources have been extended to periodontal defects [92]. Periodontitis leads to pathologic loss of periodontal ligament, cementum and alveolar bone [93]. Receptor activator of NF-κB ligand (RANKL) plays a main role in osteoclastogenesis during inflammatory bone resorption and activated T and B cells can be the source of this cytokine in periodontal disease [94]. MSCs from different origins have shown a periodontal regenerative potential in animal models without adverse effects. In particular, local implantation of periodontal ligament-derived MSCs have demonstrated their ability in the regeneration of periodontal ligament and cementum [92]. Similarly, collagen sponges containing CM from BM-MSCs have been shown to repair periodontal defects in rats 4 weeks after implantation with regeneration of alveolar bone, cementum and periodontal ligament. This CM contains a number of growth factors such as insulin-like growth factor-1, VEGF, TGF-β1 and HGF which could mediate mobilization of endogenous MSCs, angiogenesis and differentiation [95]. Interestingly, CM from BM-MSC stimulates alveolar bone augmentation prior to dental implant placement in humans without inducing an inflammatory response [96]. Implants of collagen sponges including EXOs (from HuES9.E1 cells-derived MSCs) have been shown to increase the formation of new alveolar bone in a rat model of surgically created periodontal intrabony defects. These EXOs could increase rat periodontal ligament cell migration and proliferation through CD73-mediated adenosine receptor activation of pro-survival Akt and ERK signaling pathways [97].

These studies suggest that EVs may be used for bone tissue repair and bone regeneration-related diseases avoiding drawbacks of MSC transplantation. Different approaches are feasible, for instance, EVs may be applied to pretreat autologous cell populations prior to implantation or included in biomimetic scaffolds that are used clinically [75]. Another possibility is the use of EVs from pre-conditioned MSCs or modified EVs overexpressing proteins, miRNAs and so forth, to potentiate their regenerative properties. Nevertheless, it should be taken into account that endogenous EVs can be released by BM-MSCs, osteoblasts, osteoclasts, osteocytes, endothelial cells and immune cells leading to a complex communication network which plays an important role in bone formation, repair and remodeling (reviewed in References [98,99]). Further studies are required to identify the active components and dissect the mechanisms involved in these processes as well as their modification by the therapeutic administration of MSC EVs.

## 4. Cartilage Repair and Osteoarthritis

Autologous or allogenic MSCs have demonstrated their ability for cartilage repair in vitro and in vivo, in animal studies and clinical trials. Therefore, MSCs exhibit a high therapeutic potential for the treatment of cartilage defects or OA. Different strategies have been explored to improve the regenerative properties of MSCs such as co-cultures with chondrocytes to promote MSC chondrogenesis, combination of cells with different scaffolds and so forth. [100,101]. There is increasing evidence that release of EVs is the primary way of communication in these co-cultures and they may transfer bioactive molecules between different cell types to regulate their functions [102]. MSC EVs may modulate the immune microenvironment to reduce the pro-inflammatory cell phenotype while promoting a regenerative milieu for tissue repair.

Joint injury induces the production of cytokines, chemokines and danger-associated molecules, leukocyte migration and activation of articular cells leading to catabolic processes that may degrade cartilage and bone. Therefore, pro-inflammatory cytokines IL-1β, IL-6, IL-8 and TNFα are present in synovial fluid from patients presenting with traumatic knee injury [103] and their levels are higher in the presence of osteochondral fracture [104]. It is widely accepted the relevance of pro-inflammatory mediators in the production and activation of degradative enzymes such as MMPs, a disintegrin and metalloproteinase with thrombospondin motifs (ADAMTS) and so forth. [105]. Although this pro-inflammatory response is necessary to start the repair process, if it lacks adequate control can contribute to perpetuate chronic inflammation and tissue damage leading to post-traumatic OA [106]. In a model of chronic inflammation in OA synoviocytes treated with IL-1β, it was demonstrated that EVs can be internalized by OA synoviocytes. This process could depend on the interaction of EV surface molecules such as CD44 with the hyaluronan matrix on synoviocytes. Treatment with AD-MSC-derived EXOs downregulated inflammatory mediators such as IL-6, CCL-2 and CCL-5 and these anti-inflammatory effects may be mediated by the transfer of miRNAs [107].

**Figure 3:**
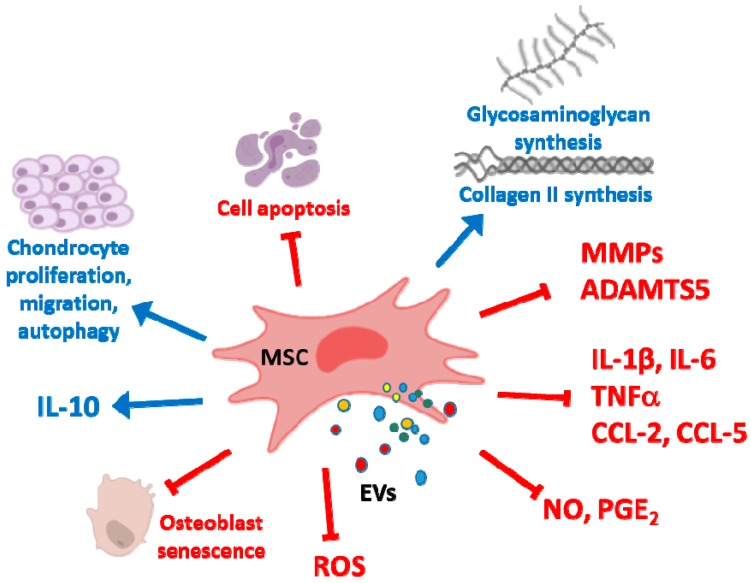
Effects of MSC EVs on cartilage repair and osteoarthritis (OA). In chondrocytes, MSC EVs downregulate pro-inflammatory mediators such as IL-1β, IL-6, IL-8, IL-17, CCL-2, CCL-5, NO and PGE_2_ as well as catabolic enzymes MMPs and ADAMTS5. Besides, these EVs enhance chondrocyte proliferation, autophagy and the synthesis of cartilage extracellular matrix. IL-10 levels are increased in OA chondrocytes and subchondral osteoblasts. In these last cells, MSC EVs also exert anti-inflammatory effects and reduce mitochondrial membrane alterations and oxidative stress to protect osteoblasts from stress-induced senescence.

EVs from human MSCs are incorporated into OA chondrocytes and exert anti-inflammatory and anti-catabolic effects (Figure 3). In the presence of TNFα stimulation, EVs downregulated the production of inflammatory mediators (IL-1α, IL-1β, IL-6, IL-8, IL-17) and cyclooxygenase-2 (COX-2) expression, as well as the release of collagenase activity whereas they promoted chondrocyte proliferation and did not affect apoptosis. These effects of EVs were dependent on the inhibition of NF-κB inhibitor α (IκBα) phosphorylation and NF-κB activation. In addition, in OA chondrocytes cultured in fibrin constructs, treatment with BM-MSC EVs improved the synthesis of main cartilage extracellular matrix components type II collagen and aggrecan and increased the expression of chondrogenic genes SRY-box 9 (SOX9) and Wnt-7A. In contrast, gene expression of hypertrophic factors runt related transcription factor 2 (RUNX2), type X collagen and alkaline phosphatase (ALP) were inhibited [108].

We have shown the protective effects of MVs and EXOs from human AD-MSCs in OA chondrocytes stimulated with IL-1β. Both types of EVs significantly reduced the production of pro-inflammatory and catabolic mediators TNFα, IL-6, MMPs, NO and PGE_2_ due to the inhibition of NF-κB and activator protein-1. The downregulation of inducible nitric oxide synthase (iNOS) would be responsible for the reduction in NO, while the inhibitory effects on gene expression of COX-2 and microsomal PGE synthase-1 determined PGE_2_ decrease. In addition, the production of IL-10 and the expression of type II collagen were enhanced. This study also showed that annexin A1 may partly mediate the chondroprotective effects of AD-MSC MVs [63]. In addition, MVs and EXOs mediate the paracrine effects of AD-MSC on OA osteoblasts. These EVs inhibited the production of inflammatory mediators IL-6 and PGE_2_, mitochondrial membrane alterations and oxidative stress to protect osteoblasts from stress-induced senescence and DNA damage while IL-10 production was enhanced [109].

Intra-articular injections of EXOs (100 μg in 100 μL of injection) from immortalized E1-MYC 16.3 human embryonic stem cells (ESCs) administered immediately after the surgery and subsequently on a weekly basis, resulted in cartilage regeneration in a model of rat osteochondral defect on the trochlear grooves of the distal femurs. The repair process was started at 2 weeks and continued at weeks 6 and 12. EXOs promoted hyaline cartilage formation with chondrocytic cells and high expression of sulfated glycosaminoglycans and type II collagen and low expression of type I collagen. Interestingly, EXOs may increase the number of chondrocytes through different mechanisms such as increased proliferation, reduced apoptosis and enhanced recruitment. The modulation of the inflammatory response may contribute to the effects of EXOs as they induced a higher infiltration of CD163^+^ regenerative M2 macrophages with respect to the inflammatory CD86^+^ M1 macrophages at the defect site and reduced the levels of synovial IL-1β and TNFα. The phosphorylation of survival pathways such as Akt and ERK through exosomal CD73 transfer mediated EXOs effects on cell migration and proliferation [110]. In this model, intra-articular injections of EXOs from HuES9 ESC-MSCs after surgery and then on a weekly basis promoted cartilage repair at 12 weeks after surgery. Interestingly, no adverse inflammatory response was observed suggesting the potential application of human MSC EXOs in an allogeneic manner [111].

In order to retain the EXOs at the cartilage defect site to prolong their effects and achieve a complete regeneration, EXOs have been incorporated into different scaffolds. Therefore, iPSC-MSC-derived EXOs have been applied incorporated in a photoinduced imine crosslinking hydrogel tissue patch. This strategy resulted in the formation of new hyaline cartilage with many resident chondrocytes covering the defect and integrated with the native cartilage in a rabbit articular cartilage defect model [112]. Implantation of a 3D printed cartilage extracellular matrix/gelatin methacrylate/EXOs (from mouse BM-MSCs) scaffold after surgery, exerted regenerative effects in a model of osteochondral defect in rabbit. This treatment seemed to improve chondrocyte mitochondrial dysfunction and cartilage formation although subchondral bone regeneration was enhanced by this scaffold independently of the presence of EXOs [113].

In spite of the fact that clinical studies using MSCs for OA have shown some promising effects long-term controlled studies with larger sample size are required (for a review, see References [114,115]). EVs from different types of MSCs have demonstrated a therapeutic effect in experimental OA. Thus, EXOs (10^8^ particles) obtained from human AD-MSCs during chondrogenic differentiation mixed with hyaluronic acid hydrogel were injected into the joint of monosodium iodoacetate (MIA) rats once a week for 3 weeks. This treatment prevented proteoglycan degradation and reduced cartilage destruction [116].

ESC-MSCs can be another relevant source of EXOs for therapeutic purposes. In the destabilization of the medial meniscus (DMM) OA model in mice, intra-articular injection of H1 human ESC-MSCs (1 × 10^6^) in mice at 4 weeks after DMM surgery significantly reduced cartilage erosion. These effects may be mainly mediated by EVs as administration of these microparticles resulted in their colocalization with collagen II-expressing chondrocytes. Thus, EXOs treatment maintained the chondrocytic phenotype by enhancing collagen type II synthesis and decreasing ADAMTS5 expression in the presence of IL-1β leading to significant reductions in cartilage destruction [117]. EXOs derived from infrapatellar fat pad MSCs enhanced chondrocyte autophagy partly through mammalian target of rapamycin (mTOR) inhibition, while promoted chondrocyte proliferation and extracellular matrix synthesis protecting against cartilage degradation in the DMM model. The delivery of miR-100-5p to chondrocytes may partly mediate the protective effects of these EXOs [118]. In the collagenase model in mice, intra-articular administration of human iMSC-EXOs or synovial membrane MSC EXOs (10^10^/mL, 8 μL on days 7, 14 and 21) reduced cartilage degradation assessed on day 28 with improvement in macroscopic and histological scores. Both treatments also enhanced the in vitro proliferation and migration of human chondrocytes [119].

On the other hand, MSCs are a promising therapy for the treatment of temporomandibular joint OA [120] which is characterized by progressive cartilage degradation, subchondral bone erosion and chronic pain [121]. Articular injection of autologous BM-MSCs led to cartilage regeneration and retarded the progressive cartilage and subchondral cancellous bone lesions in a partial disc resection model of temporomandibular joint OA in rabbits. In vitro chondrogenic induction of MSCs could enhance these protective effects [122]. Intra-articular administration (100 μg in 50 μL on a weekly basis for 2, 4 or 8 weeks) of EXOs from immortalized E1-MYC 16.3 ESC-MSCs suppressed inflammation and pain, reduced fibrosis and promoted matrix repair of cartilage and subchondral bone after induction of temporomandibular joint OA in rats by MIA injection. In addition, EXOs treatment enhanced cellular proliferation and reduced apoptosis and the expression of genes associated to inflammation, apoptosis, fibrosis and pain. Furthermore, EXOs protected against extracellular matrix degradation through the upregulation of tissue inhibitor of metalloproteinase (TIMP)-2 which inhibits MMPs and the upregulation of ADAMTS5. The pathways involved in these effects were examined in vitro in rat temporomandibular joint condylar chondrocytes stimulated with IL-1β. The results demonstrated a role for Akt, ERK and adenosine monophosphate-activated protein kinase (AMPK) activation induced by adenosine [123]. CM and EXOs from human exfoliated deciduous teeth MSCs inhibit gene expression of IL-6, IL-8 and ADAMTS5 as well as gene and protein expression of MMP-1, MMP-9 and MMP-13 in human temporomandibular chondrocytes stimulated with IL-1β. These EXOs are enriched in miR-100-5p which may be responsible for the observed effects through directly targeting mTOR [124].

Modification of EV composition can improve their regenerative properties. Different strategies have been investigated such as pre-stimulation of MSCs with LPS, inflammatory cytokines, growth factors or NO, hypoxia preconditioning, cellular reprogramming of MSCs and so forth, (for a review, see Reference [125]). Recently, overexpression of miRNAs has been revealed as a promising strategy in cartilage protection. miR-320 expression is reduced in OA cartilage compared with normal tissue and it promotes chondrogenesis and downregulates MMP-13 in mouse chondrocytes stimulated with IL-1β [126]. Therefore, overexpression of miR-320c in human BM-MSCs led to EXOs enriched in this miRNA which were more effective than unmodified EXOs to downregulate MMP-13 and enhance SOX9 and type II collagen expression, cell motility and proliferation in human OA chondrocytes [127]. Besides, BM-MSC-derived exosomal miR-92a-3p regulates cartilage homeostasis by acting as a Wnt-5A inhibitor [128]. Stimulation of MSCs with TGF-β1 increases miR-135b expression in EVs leading to the negatively regulation of specificity protein 1 (SP1) and the enhancement of chondrocyte proliferation [129]. In experimental OA, miR-140-5p exerts protective effects against synovial injury by inhibiting inflammatory reactions and apoptosis of synoviocytes [130]. Transfection of miR-140-5p in human synovial MSC led to EVs enriched in this miRNA which enhanced human chondrocyte proliferation and migration in vitro. In vivo experiments using a rat surgical OA model showed the efficacy of this treatment after intra-articular injection of 100 μL (10^11^ particles/mL) on the first day of every week from the 5th to the 8th week after surgery. Histological analysis of samples obtained 12 weeks after surgery indicated a preventive effect on joint degradation and loss of cartilage extracellular matrix [131].

MSC EXOs also contain the lncRNA KLF3-AS1 which inhibits chondrocyte apoptosis and promotes cartilage repair in OA models [132]. Chondroprotective effects were related to miR-206 sponging to facilitate G-protein-coupled receptor kinase-interacting protein 1 (GIT1) expression [133]. In OA there is a downregulation of hsa_circ_0000077 (circ77) in cartilage while overexpression of this circular RNA in chondrocytes protects against cartilage degeneration. Similarly, EVs from circ77-overexpressed synovial MSCs augmented chondrocyte proliferation and the expression of SOX9, type II collagen and aggrecan thus counteracting the effects of IL-1β [134].

Another strategy may be the obtention of EVs from bile acids-treated MSCs which enhanced SOX9 and type II collagen expression in chondrocytes whereas the hypertrophic marker MMP-13 was decreased [135]. EV-mimetic vesicles have been obtained by serial extrusions of human AD-MSCs through filters. Injection of these vesicles into the joints of MIA rats improved pain and joint alterations at day 24 [136]. In addition, MSCs are also the source of nanoghost particles derived from the cytoplasmic membrane and targeting human chondrocytes. These particles are effective to reduce the expression of inflammatory and catabolic markers thus opening a number of possibilities for cartilage regeneration therapy [137].

## 5. Intervertebral Disk Degeneration

Intervertebral disk degeneration (IVD) leads to low back and neck pain besides disability. In IVD there is an increase in the levels of pro-inflammatory cytokines TNFα, IL-1α, IL-1β, IL-6 and IL-17 which mediate chemokine production, macrophage infiltration, phenotype alterations, premature senescence and extracellular matrix degradation [138,139,140]. Oxidative stress may also play a role in the pathogenesis of IVD through the induction of apoptosis and calcification of the cartilage endplate cells [141]. MSCs transplantation represents an interesting strategy for disc regeneration as they can differentiate into nucleus pulposus-like cells [142] and promote cell proliferation and inhibition of apoptosis [143]. In IVD there is a reduced expression of Fas ligand (FasL) in these cells, which alters their interaction with immune cells. Therefore, MSCs expressing FasL may reinforce immune privilege in nucleus pulposus cells as another mechanism for disc regeneration [144].

MSC-derived EVs represent an appropriate alternative to the use of MSCs in IVD as they are more stable than these cells which are strongly influenced by the inflammatory microenvironment [145]. Several studies have demonstrated the incorporation and actions of MSC-EXOs in nucleus pulposus cells [143,146]. BM-MSCs EXOs promote nucleus pulposus cell proliferation and restore the imbalance between catabolism and anabolism with downregulation of MMP-1 and MMP-3, upregulation of TIMP-1 and improvements in extracellular matrix. In turn, nucleus pulposus cell-derived EXOs can induce the migration and differentiation of BM-MSCs into nucleus pulposus-like cells in vitro [147,148]. These EXOs attenuated endoplasmic reticulum stress induced by advanced glycation end products (AGEs) and apoptosis, by activating Akt and ERK signaling in human nucleus pulposus cells. In vivo studies have shown that BM-MSCs EXOs inhibit the progression of IVD in a rat tail model [146]. These protective effects may be mediated in part by the transfer of exosomal miR-21 which restrains phosphatase and tensin homolog (PTEN) leading to the activation of the PI3K/Akt pathway [143].

## 6. Skeletal Muscle and Tendon Repair

MSCs may have applications in the treatment of human muscular disorders due to their ability to differentiate into functional skeletal muscle cells [115,149]. Nevertheless, there is limited preclinical evidence demonstrating the ability of MSC EVs to accelerate the repair of muscle tissues. The secretome of human amniotic fluid stem cells and its EVs have been shown to enhance angiogenesis leading to muscle regeneration [150]. Administration (i.v.) of the secretome or the EV fraction of human AD-MSCs or BM-MSCs increased skeletal muscle regeneration following acute damage induced by cardiotoxin I in mice. The enhancement of myogenesis and angiogenesis mediated by miRNAs such as miR-494 would determine the regenerative activity [151,152]. In the mouse cardiotoxin I model, injury results in necrotic muscle fibers with an important mononuclear cell infiltrate. The inflammatory response was reduced by EV treatment, as shown by a reduction in IL-16/IL-10 ratio at day 1 and 2 after the lesion. In addition, M1 markers significantly decreased and M2 markers significantly increased at the last time point indicating the ability of EVs to switch macrophages into the anti-inflammatory and healing phenotype which mediates the activation of myogenic precursors. Hypoxia pre-conditioning of MSCs induced pro-angiogenic factors and the secretion of EVs with increased expression of VEGF-A and platelet and endothelial cell adhesion molecule 1 (PECAM1) as well as of miRNAs such as miR-126. In addition, EVs from hypoxia-conditioned AD-MSCs increased the expression of CCL-2 at day 2, which plays an important role in macrophage recruitment leading to the upregulation of myogenic markers such as Pax7 and MyoD and new multinucleated muscle fibers [55]. In addition, results from a rat model of massive rotator cuff tear using EXOs (10^11^ in 20 μL of saline) from AD-MSCs injected into two locations of the supraspinatus muscle indicate that this treatment delayed the progression of degenerative changes. Thus, EXOs from AD-MSCs may represent a cell-free approach to prevent atrophy and muscle degeneration associated to torn rotator cuffs [153].

EVs also have the potential to enhance tendon repair via paracrine regulation of the inflammatory response to injury [154]. In a model of Achilles tendon healing after unilateral surgical transection, AD-MSC EVs were loaded to the surface of a collagen sheet which was applied around the repair site. This treatment attenuated inflammatory cell migration and NF-κB activation whereas the anabolic response was facilitated leading to tendon matrix regeneration [154]. Another possible strategy consists of polarizing macrophages to an M2 phenotype by incubating these cells with EVs from human BM-MSCs. These M2 macrophages may increase angiogenesis and repair in the above model of tendon transection [155].

## 7. Conclusions

There is a need for efficacious and tolerable treatments which could be developed as novel therapeutics for musculoskeletal disorders. In this context, preclinical studies have shown promising results that will provide the basis for therapeutic applications of EVs. Increasing evidence has shown that EVs play an important role in mediating the effects of MSCs and they may have some advantages compared with cell therapy. Whereas MSCs might change their functions by the influence of the environmental milieu of the recipient [156], EVs treatments may lead to more predictable effects and a better control of treatment. Besides, the use of EVs would avoid some risks of MSC therapy such as alterations of cell phenotype or thrombosis [157,158].

A critical question to be addressed is the heterogeneity of separation protocols and preparations of EVs. For future translation into the clinic, all these processes must be standardized. In this regard, the Minimal Information for Studies of EVs (MISEV2018) [31] recommends specific criteria for definition and classification of EVs although it does not provide guidance on functional testing of biological activity. Progress in the knowledge of properties and applications of EVs requires the characterization of their preparations by physical, biochemical and functional attributes using reproducible and standardized assays [159].

In addition, further research is needed to optimize the effects of MSC EVs as well as to know the active components responsible for their actions, underlying mechanisms, pharmacokinetic aspects and safety of this approach. Future studies will provide the necessary insight to develop MSC EVs for clinical applications.
